# How (Ultra‐)Rare Gene Variants Improve Our Understanding of More Common Autoimmune and Inflammatory Diseases

**DOI:** 10.1002/acr2.70003

**Published:** 2025-02-18

**Authors:** Alexandre Belot, Maud Tusseau, Jade Cognard, Sophie Georgin‐Lavialle, Guilaine Boursier, Christian M. Hedrich

**Affiliations:** ^1^ Centre International de Recherche en Infectiologie, University of Lyon, Inserm U1111, Université Claude Bernard Lyon 1, Centre National de la Recherche Scientifique, UMR5308, École normale supérieure de Lyon, National Referee Centre for Rheumatic and Autoimmune and Systemic Diseases in Children, and Hôpital Femme Mère Enfant, Hospices Civils de Lyon, Lyon, France, and French National Reference Center of Autoinflammatory Diseases and Amyloidosis Lyon France; ^2^ Centre International de Recherche en Infectiologie, University of Lyon, Inserm U1111, Université Claude Bernard Lyon 1, Centre National de la Recherche Scientifique, UMR5308, École normale supérieure de Lyon, National Referee Centre for Rheumatic and AutoImmune and Systemic Diseases in Children, and Hôpital Femme Mère Enfant and Groupement Hospitalier Est, Hospices Civils de Lyon, Lyon, France, and French National Reference Center of Autoinflammatory Diseases and Amyloidosis Paris France; ^3^ American Memorial Hospital, Centre Hospitalier Universitaire Reims, Reims Champagne‐Ardenne University Reims France; ^4^ French National Reference Center of Autoinflammatory Diseases and Amyloidosis, Paris, France, and Sorbonne Université, Hôpital Tenon, DMU 3ID, AP‐HP Paris France; ^5^ French National Reference Center of Autoinflammatory Diseases and Amyloidosis, Paris, France, and Centre Hospitalier Universitaire Montpellier, University of Montpellier Montpellier France; ^6^ Institute of Life Course and Medical Sciences, University of Liverpool and Alder Hey Children's NHS Foundation Trust Liverpool United Kingdom

## Abstract

The aim of this study was to explore the impact of rare and ultra‐rare genetic variants on the understanding and treatment of autoimmune and autoinflammatory diseases with a focus on systemic lupus erythematosus (SLE) and Behçet syndrome. This review summarizes current research on the monogenic causes of SLE and Behçet syndrome, highlighting the various pathways that can be responsible for these unique phenotypes. In monogenic SLE, the identification of complement and DNASE1L3 deficiencies has elucidated mechanisms of apoptotic body accumulation and extracellular nucleic acid sensing. Type I interferonopathies underline the specific role of DNA/RNA sensing and the interferon overexpression in the development of systemic autoimmunity. Other significant genetic defects include Toll‐like receptor hypersignaling and JAK/STATopathies, which contribute to the breakdown of immune tolerance. To date, genetic defects directly affecting B and T cell biology only account for a minority of identified causes of monogenic lupus, highlighting the importance of a tight regulation of mechanistic target of rapamycin and RAS (Rat sarcoma GTPase)/MAPK (mitogen‐activated protein kinase) signaling in lupus. In Behçet syndrome, rare variants in *TNFAIP3, RELA*, and *NFKB1* genes have been identified, underscoring the importance of NF‐κB overactivation. Additional monogenic diseases such as *ELF4, WDR1* mutations and trisomy 8 further illustrate the genetic complexity of this condition. Observations from genetic studies in SLE and Behçet syndrome highlight the complexity of systemic inflammatory diseases in which distinct molecular defects caused by single‐gene mutations can promote lupus or Behçet syndromes, often unrecognizable from their genetically complex “classical” forms. Insights gained from studying rare genetic variants enhance our understanding of immune function in health and disease, paving the way for targeted therapies and personalized medicine.

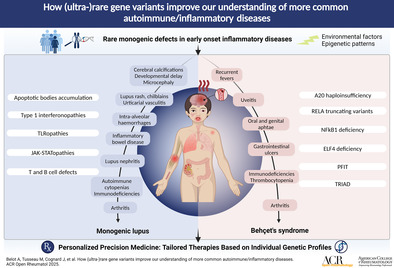

## Introduction

Systemic inflammatory diseases, encompassing both autoimmune and autoinflammatory conditions, largely remain of unknown origin. They can be broadly categorized based on their genetic background into monogenic and polygenic (or genetically complex) diseases.^1^, ^2^, ^3^, ^4^ The monogenic model refers to rare diseases caused by large‐effect‐size mutations in a single gene that, through their strong impact on gene function, most commonly result in severe and early‐onset conditions.^5^ These rare variants, although individually rare, provide significant insights into the mechanisms of immune (dys)regulation and tolerance in humans. Among the first described, large‐effect‐size variants in complement genes were associated with early‐onset systemic lupus erythematosus (SLE), highlighting the critical role of classical complement pathway in maintaining immune tolerance.^6^

In contrast, the polygenic model involves the interplay of multiple genetic variants, each contributing a small effect to the overall disease risk.^5^ In addition to gene variants (so‐called risk alleles), environmental impacts contribute to disease expression, in many cases through the induction of epigenetic remodeling and associated dysregulation of gene expression.^3^, ^7^, ^8^ Genetically complex polygenic traits account for more common inflammatory diseases such as rheumatoid arthritis or Crohn disease. Genome‐wide association studies and epigenetic profiling have identified numerous loci associated with these diseases, illustrating the complex chromatin architecture underlying polygenic diseases.^3^, ^9^ Most commonly, these disease‐associated genetic variants are noncoding single‐nucleotide polymorphisms with limited effects on protein expression or function.^10^ Exceptions were identified in the class I or class II HLA loci showing strong associations. The HLA‐B27 allele, despite being relatively common in the population (approximately 8% of the European population), significantly increases the risk of developing ankylosing spondylitis and other spondyloarthropathies.^11^ The HLA‐B27 allele accounts for approximately 20% of the genetic risk for these diseases, demonstrating how frequent variants on a population level can play a major role in disease susceptibility.^12^

The study of monogenic susceptibility to infectious diseases, pioneered by Jean‐Laurent Casanova, has furthered our understanding of complex diseases such as infections.^13^, ^14^ Single‐gene variants with large effect size can predispose individuals to severe infections (eg, with mycobacteria and/or viruses).^15^, ^16^ These findings not only elucidate the genetic basis of infectious diseases but also provide a framework for understanding nonredundant immune functions in humans. Through the identification of critical immune pathways disrupted by rare variants, potential therapeutic targets applicable to a broader range of conditions can be identified.

In summary, identification of rare variants in early‐onset inflammatory diseases improved the current understanding of the genetic landscape of several inflammatory diseases. Disease‐causing gene variants offer profound insights into specific immune mechanisms, paving the way to personalized medicine, in which genetic information guides the diagnosis, risk stratification, and outcome prediction as well as treatment of inflammatory diseases. This review explores the role of rare variants in early‐onset inflammatory syndromes to understand complex diseases focusing on SLE and Behçet syndrome as examples.

## Definition of (ultra‐)rare variants

Rare and ultra‐rare genetic variants are defined by their low frequency in the general healthy population, typically with a minor allele frequency of <1% for rare variants and <0.1% for ultra‐rare variants.^17^ “Novel variants” have not been identified in current population databases such as Genome Aggregation Database (gnomAD) version 4, comprising >730,000 of exomes and 75,000 genomes from unrelated individuals.^18^ Verification of the potential functional impact of a novel variant can be complex. To explore the functional relevance of newly identified variants, the first step is usually to start with in silico analysis. This involves using computational tools and databases to predict the potential impact of these variants on protein function and disease. Metapredictor tools, such as Rare Exome Variant Ensemble Learner (REVEL),^19^ MetaSVM,^20^ Combined Annotation Dependent Depletion (CADD),^21^ and, more recently, AlphaMissense,^22^ combining structural information from AlphaFold^23^ with evolutionary conservation, aid with the prioritization of variants based on their predicted pathogenicity. Online applications bring together most of them and other useful data such as MobiDetails.^24^

However, in silico predictions alone are not sufficient to reliably confirm the biologic relevance of novel variants.^25^ Therefore, functional assays are essential to validate their effects in a biologic context. The definition of functional impact relies on the quantification of protein/RNA expression, cellular functional models, experiments using primary cells, and in vivo animal models (eg, knock‐in mice).^26^

The first genetic discovery, beyond complement deficiencies, was the identification of *MEFV* (Mediterranean fever) gene variants in patients with familial Mediterranean fever (FMF) in 1997.^27^, ^28^ Biallelic variants in exon 10 of *MEFV* are associated with FMF, a hereditary cause of recurrent fever. Over the past 25 years, numerous functional studies have shown the role of pyrin as a critical sensor for intracellular danger signals via modifications of the cytoskeleton, leading to the activation of caspase‐1, and in turn, it processes and activates proinflammatory cytokines such as interleukin (IL)‐1b.^29^ Patients with FMF can display additional inflammatory diseases such as IgA‐associated vasculitis or HLAB27‐negative spondyloarthropathies, psoriasis, or hidradenitis suppurativa, providing clues to explore these more frequent inflammatory diseases.^30^, ^31^ Thus, genetic studies of rare monogenic diseases have been instrumental in uncovering the mechanisms of dysimmunity. Here, we highlight the model of rare variants to elucidate complex diseases with two diseases: SLE, a paradigm of autoimmune diseases, and Behçet disease, recently redefined as Behçet syndrome.^32^, ^33^

## Ultra‐rare variants and monogenic SLE

**Figure 1:**
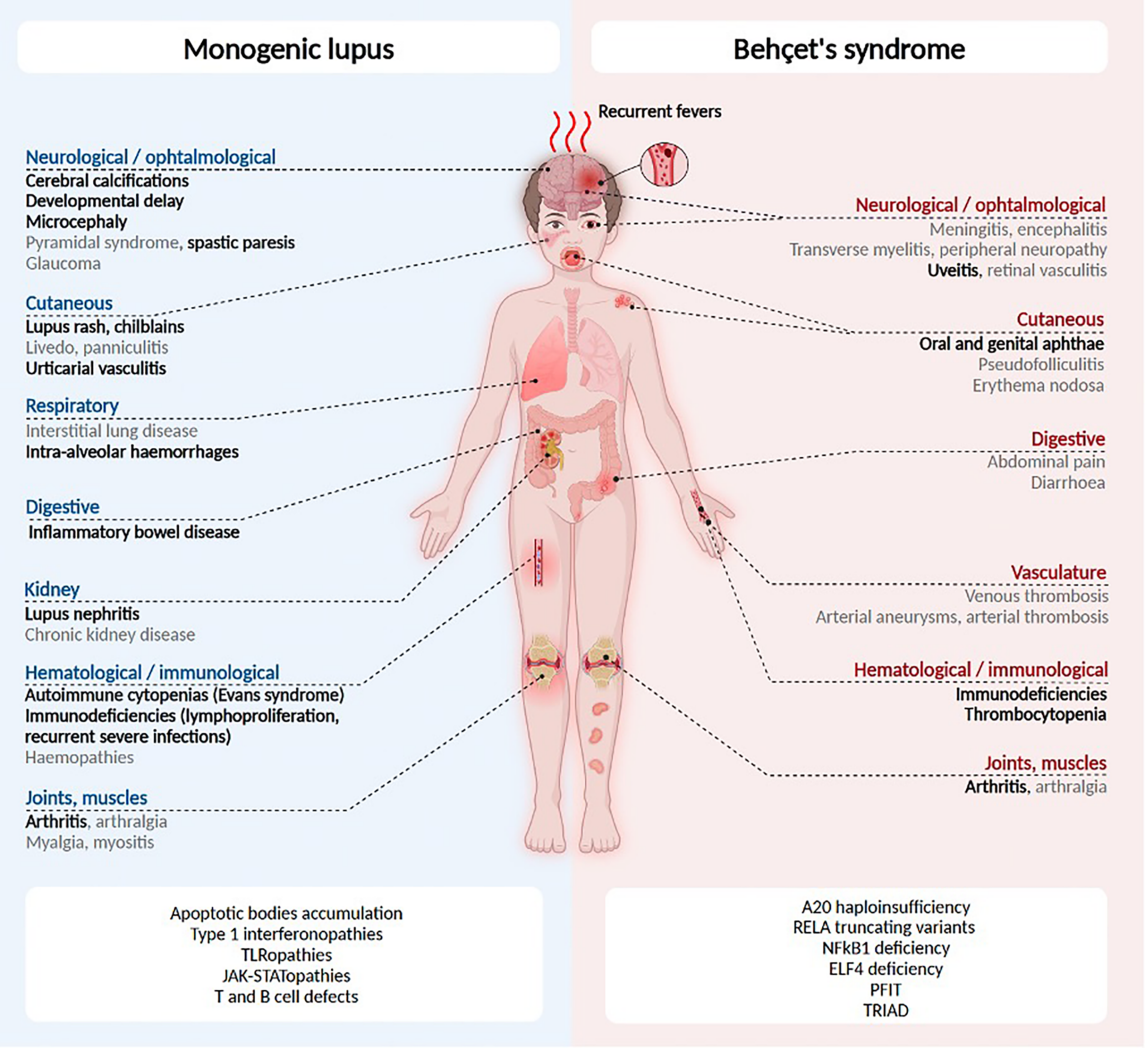
Monogenic causes and clinical phenotypes of lupus and Behçet syndrome. ELF, E74‐like ETS transcription factor; PFIT, periodic fever, immunodeficiency, and thrombocytopenia; RELA, v‐rel reticuloendotheliosis viral oncogene homolog A; TLR, Toll‐like receptor; TRIAD, trisomy 8–associated autoinflammatory disease.

SLE is a clinically and pathophysiologcally complex systemic inflammatory disease that can affect every organ of the human body (Figure 1).^32^ The majority of patients with SLE carry relatively common variants (so‐called risk alleles) that are not strong enough to cause disease individually. Thus, a combination of risk alleles and/or additional factors, accumulated over time (including hormonal changes, exposure to infections medication, etc), must co‐occur to allow disease expression.^34^ Conversely, a small proportion of patients carry (ultra‐)rare gene variants with large effect size that are strong enough to confer disease.^34^, ^35^, ^36^

Approximately 30 years after the discovery of complement deficiencies, the monogenic model of SLE has been conceptualized in the early 2010s.^37^ High‐throughput gene sequencing has since enabled the identification of a constantly growing number of genes responsible for monogenic lupus affecting several immune pathways,^35^, ^36^ highlighting the heterogeneity of genetic defects leading to systemic autoimmunity. The identification of monogenic SLE and SLE‐like disease is of clinical relevance because it associates not only with earlier disease during childhood but also distinct clinical phenotypes and disease trajectories that can differ from “classic” genetically complex forms of SLE (Figure 1). Indeed, children carrying rare and predicted pathogenic variants have been suggested to more commonly develop central nervous system (CNS) disease when compared to the remaining cohort of patients with juvenile‐onset SLE.^38^

### Accumulation of apoptotic bodies

One of the first identified groups of monogenic causes of systemic inflammatory disease were complement deficiencies in the late 1970s.^6^, ^39^ The complement system comprises a group of plasma proteins that play a crucial role in the innate immune defense. Activated complement enhances antibody‐mediated pathogen opsonization, facilitating the removal of bacteria, infected cells, immune complexes, and apoptotic bodies.^40^ Defects of component components of both the classical and lectin pathways can cause accumulation of apoptotic bodies, leading to the production of autoantibodies and inflammation‐mediated tissue damage that amplifies autoantibody formation and immune complex deposition through tissue damage and the release of nuclear components to the extracellular space.^41^ Complement deficiencies, particularly in the early components (C1–C4), are associated with monogenic forms of lupus.^42^, ^43^

Homozygous complement C1q deficiency, caused by loss‐of‐function mutations in the *C1QA/B/C* genes, is linked to early‐onset SLE or SLE‐like disease in nearly 90% of affected individuals. Only a few dozen patients have been reported.^42^ Deficiencies of C1r and C1s are even more rare and present with a lower risk (around 60%) of developing SLE or lupus‐like disease. These defects are, however, primarily characterized by immunodeficiency, with over 80% of patients experiencing recurrent severe infections.^43^

Complete complement C4 deficiency, affecting both the *C4A* and *C4B* genes, is extremely rare and results in lupus or autoimmune renal disease in 80% of patients. Partial deficiencies of C4A or C4B are more common. Partial C4A deficiency has a prevalence of about 1:1,000 individuals and is a known risk factor for lupus but is not formally considered a monogenic disease.^44^

Complement C2 and C3 deficiencies are less penetrant. Approximately 10% to 20% of individuals with these deficiencies develop SLE or autoimmune diseases, often accompanied by recurrent infections. Complement C2 deficiency has an estimated prevalence of 1:20,000 individuals, whereas <50 patients with C3 deficiency have been reported.^43^

Management of complement deficiencies is challenging because no supplementation strategies are available to restore plasmatic complement level.^45^ To reduce the expression of autoantibodies and the number of autoreactive B cells, B cell depletion has been tried and may control disease in a subset of patients.^46^ In patients who were treatment refractory, the inhibition of JAKs reduced interferon (IFN) expression and associated systemic disease activity.^47^

In the same pathway, another gene, *DNASE1L3*, has been associated with a lupus phenotype,^48^ with about 50 patients reported in the literature.^49^ The secreted DNASE1L3 serum enzyme plays a crucial role by degrading antigenic forms of cell‐free DNA derived from apoptotic bodies.^50^ Biallelic mutations in *DNASE1L3* commonly lead to SLE‐like disease with nephritis and hypocomplementemic urticarial vasculitis (McDuffie syndrome). Furthermore, DNASE1L3 deficiency is associated with antineutrophil cytoplasmic antibody positivity and, rarely, alveolar hemorrhage and inflammatory bowel disease.^49^ Mouse models suggest *Dnase1l3* deficiency leads to the development of antibodies to double‐stranded DNA and chromatin, resulting in a lupus‐like phenotype. Notably, these manifestations are abolished by the deletion of myeloid differentiation factor 88, indicating the role of Toll‐like receptor (TLR) signaling in this model in which the extracellular function of DNase1L3 is preventing TLR activation and subsequent autoimmune responses.^50^ The type I IFN signature in patients with DNASE1L3 deficiency is generally lower when compared to other “canonical” interferonopathies.^49^ Furthermore, likely as a result of tissue damage during infections, IFN expression and disease activity wax and wane in individual patients with DNASE1L3 deficiency.^49^ In the absence of clinical trials or large cohorts, treatment of DNASE1L3 deficiency is challenging; future developments of enzyme‐replacement therapies may offer new options.^45^

**Figure 2:**
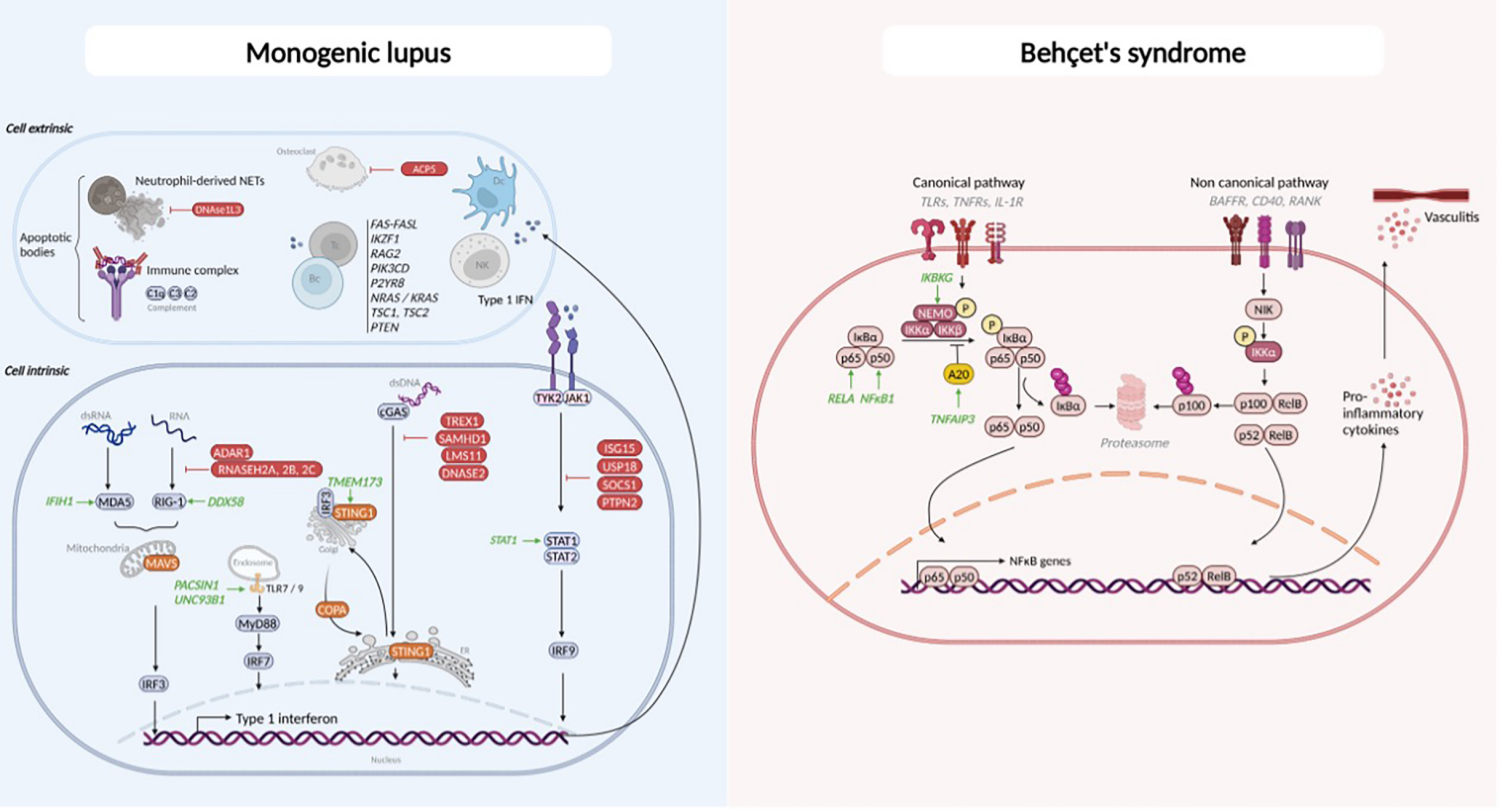
Pathways involved in monogenic systemic lupus erythematosus and Behçet syndrome. ACP, acid phosphatase; ADAR, adenosine deaminases that act on RNA; BAFFR, B cell–activating factor receptor; Bc, B cell; cGAS, cyclic guanosine monophosphate–AMP synthase; COPA, coatomer subunit alpha; Dc, dendritic cell; dsDNA, double‐stranded DNA; dsRNA, double‐stranded RNA; ER, endoplasmic reticulum; IFN, interferon; IL‐1R, interleukin‐1 receptor; IRF, IFN regulatory factor; ISG, IFN‐stimulated gene; MAVS, mitochondrial antiviral signaling protein; MDA, melanoma differentiation–associated protein; MyD, myeloid differentiation factor; NEMO, NF‐κB essential modulator; NET, neutrophil extracellular trap; NIK, NF‐κB–inducing kinase; NK, natural killer; PTPN, protein tyrosine phosphatase nonreceptor; RelB, v‐rel avian reticuloendotheliosis viral oncogene homolog B; RIG, retinoic acid–inducible gene; SOCS, suppressor of cytokine signaling; STING, stimulator of IFN genes; Tc, T cell; TLR, Toll‐like receptor, TNFR, tumor necrosis factor receptor; TREX, three‐prime repair exonuclease; USP, ubiquitin‐specific protease.

Notably, both C1Q and DNASE1L3 can be the target of autoantibodies in patients with genetically complex “classic” SLE and are associated with a higher risk of lupus nephritis. Antibodies against C1q are present in 20% to 50% of patients with lupus, whereas anti‐DNASE1L3 antibodies are found in 30% of patients.^51^, ^52^, ^53^ Thus, whether congenital or acquired, deficiency of molecules involved in the elimination of extracellular nucleic acids and immune complexes promote systemic autoimmunity (Figure 2).

### Type I interferonopathies

Type I IFNs are crucial cytokines in the immune response against viral infections, regulating both innate and adaptive immunity.^54^ The first interferonopathy, Aicardi–Goutières syndrome (AGS), was described in 1984.^55^ Interferonopathies are Mendelian disorders involving genes related to nucleic acid sensing (eg, *IFIH1*, *RIGI*), signaling (eg, *STING1*, *COPA*), and metabolism (eg, *RNASEH2A/B/C*, *TREX1*, *ACP5*, *DNASE2*).^56^

Early reports linked AGS with infantile encephalopathy with cerebral calcifications^55^ in the presence of disease‐causing mutations in *TREX1*, encoding for the three‐prime repair exonuclease (TREX) 1. Disease‐associated loss‐of‐function mutations in *TREX1* result in the accumulation of nucleic acids in the cytoplasm and subsequent constitutive up‐regulation of IFN expression.^57^ Epidemiologically, AGS due to *TREX1* mutations accounts for 25% of patients with AGS, with 60% of these patients showing autoimmune features.^58^

Clinically, interferonopathies present with neurologic symptoms, cutaneous vasculitis, and additional SLE‐like phenomena.^59^, ^60^ Additionally, more recently recognized interferonopathies include the following:

- Familial chilblain lupus is a cutaneous disease associated with increased IFN expression and the presence of SLE‐like symptoms and autoantibodies. It has been associated with *SAMHD1*, *TREX1*, or *STING1* mutations.^61^, ^62^
- Stimulator of IFN genes (STING)‐associated vasculopathy with onset in infancy (linked to *STING1* mutations)^61^, ^63^, ^64^, ^65^, ^66^ was first described in 2014. It is a rare systemic inflammatory disease characterized by severe skin and lung involvement, partially resembling SLE.
- The autosomal‐dominant immune dysregulatory coatomer subunit alpha (COPA) syndrome involves interstitial lung disease and inflammatory arthritis.^67^
- Spondyloenchondrodysplasia is a skeletal dysplasia associated with immune dysregulation and increased IFN expression.^67^, ^68^, ^69^, ^70^, ^71^
- DNASE2 deficiency is associated with increased IFN expression, cytopenia, and glomerulonephritis.^72^

Because pathologic IFN expression is a hallmark of these diseases, individualized and target‐directed treatment promises to be a promising strategy. Notably, the use of JAK inhibitors reduces IFN expression and peripheral symptoms in AGS and other interferonopathies. However, their effects on CNS involvement and neurodisability are limited, which may be linked to reduced penetrance into the cerebrospinal fluid.^73^ New treatment strategies (eg, IFN receptor [anifrolumab]– and type I IFN–directed blocking antibodies or the reduction of cytoplasmic nucleic acids through reverse‐transcriptase inhibitors) may overcome these limitations, but data are limited (Figure 2).^74^, ^75^, ^76^

### Endosomal TLR hypersignaling (TLRopathies)

Recently, *TLR7* gain‐of‐function variants have been identified in a small number of patients with SLE or antiphospholipid syndrome with or without neurologic symptoms.^77^, ^78^, ^79^ Reported gene variants increase the receptor's affinity for guanosine, leading to hypersensitive TLR7 signaling and enhanced B cell survival, finally contributing to B cell–mediated autoimmunity. The development of autoreactive B cells in these mutations occurs at immature stages of B cell development, independent of germinal centers.^77^

A rare loss‐of‐function variant in the gene encoding for protein kinase C and casein kinase substrate in neurons protein 1 (*PACSIN1*), acting downstream of TLR7, has been identified.^80^ This variant leads to impaired tumor necrosis factor receptor–associated factor (TRAF) 4–mediated inhibition of TLR7, and TRAF6‐mediated activation and IFN expression, contributing to the autoimmune response. Mutations in the gene encoding for the UNC93B1 chaperone protein (*UNC93B1*), which regulates subcellular trafficking of TLR3, 7, 8, and 9 as well as their activation status, have been reported in four patients with SLE‐like disease. These mutations result in diminished interaction with TLR7, causing TLR7 hyperactivation and autoimmune phenomena^81^, ^82^, ^83^, ^84^ through increased NF‐κB and IFN activation as well as B cell autoimmunity. B cell–targeted therapy may be of interest, and in the future, directly targeting TLR7 might be even more beneficial (Figure 2).^45^

### Excessive JAK/STAT signaling (JAK/STATopathies)

The JAK/STAT signaling pathway is crucial for immune activation and homeostasis.^85^ Hyperactivation of this pathway can lead to SLE due to cytokine hypersensitivity. The suppressor of cytokine signaling (SOCS) 1 is a cytokine‐induced inhibitor of JAK1, JAK2, TYK2, and STAT1.^86^ Loss‐of‐function mutations in *SOCS1* can result in SLE, psoriasis, and Evan syndrome.^87^ Similarly, mutations in the protein tyrosine phosphatase nonreceptor type 2 encoding *PTPN2* gene, another negative regulator of JAK/STAT, are associated with inflammatory bowel disease, immunodeficiency, SLE, and Evan syndrome.^88^, ^89^ Last, gain‐of‐function variants in *STAT1* have been reported in patients with chronic mucocutaneous candidiasis and bacterial infections, with some also developing autoimmune diseases, such as SLE.^90^ In these defects, treatment by JAK inhibitors have shown promising results (Figure 2).^45^, ^91^

### T and B cell defects

SLE is characterized by the presence of autoantibodies and activated T cells, indicating a breakdown in adaptive immunity and B cell tolerance.^92^, ^93^, ^94^ Autoimmune lymphoproliferative syndrome, caused by mutations in *FAS* or *FASLG*, highlights the role of apoptosis in sustaining self‐tolerance and preventing autoimmunity, but the phenotype is essentially a lymphoproliferative disease with autoimmune cytopenia.^95^ Other rare genetic defects associated with SLE‐like phenotype affect *IKZF1* (encoding for the DNA‐binding protein Ikaros, which is involved in B cell function and regulation^96^), *RAG2* (encoding for the recombination activating gene 2, which is involved in lymphocyte maturation), and phosphatidylinositol‐4,5‐bisphosphate 3‐kinase catalytic subunit delta (*PIK3CD*); all of which are frequently linked with infections and lymphoproliferation.^36^, ^97^ Patients with PKC‐δ deficiency present with lymphoproliferation, lupus nephritis, and moderate to severe immunodeficiency.^98^, ^99^, ^100^ This deficiency also leads to susceptibility to infections due to defective reactive oxygen species (ROS) production.^101^ Because of the activation of mitochondrial ROS responses, the use of mechanistic target of rapamycin (mTOR) inhibitors may be particularly helpful in this genotype.^102^, ^103^ Notably, mTOR plays a key role in regulating mitochondrial function and ROS production. Thereby, mTOR links cellular metabolism and immune responses, in which mTOR increases mitochondrial ROS production, contributing to lymphocyte activation, differentiation, and survival.^104^, ^105^, ^106^ In addition, tuberous sclerosis, a genetic disease caused by mutations in the tuberous sclerosis complex 1 (*TSC1*) or *TSC2*, or Cowden syndrome, which results from mutations in the tumor suppressor phosphatase and tensin homolog (*PTEN*), leads to constitutive activation of the mTOR pathway. Both diseases have been reported in association with SLE and/or SLE‐like phenotypes.^107^, ^108^, ^109^, ^110^, ^111^

RASopathies, caused by germline mutations in the RAS (Rat sarcoma GTPase)/MAPK (mitogen‐activated protein kinase) pathway, predispose to autoimmunity, including SLE.^104^, ^105^, ^106^, ^107^, ^108^, ^112^ RAS‐associated autoimmune leukoproliferative disorder results from somatic mutations in neuroblastoma RAS viral oncogene homolog (*NRAS*) or Kirsten rat sarcoma (*KRAS*), both elements of the RAS/MAPK pathway, causing selective immune abnormalities without overall developmental defects.^113^ Recent studies have identified new mechanisms of B cell tolerance breakdown. Variants in the *P2RY8* gene encoding for the P2Y purinoceptor 8 associate with SLE and SLE‐related antiphospholipid syndrome by preventing the expansion of DNA‐reactive B cells within the germinal center, favoring their negative selection (Figure 2).^114^, ^115^

## Ultra‐rare variants in Behçet syndrome and Behçet‐like disease

Behçet syndrome is a rare systemic inflammatory disease with heterogenous clinical presentations, including recurrent oral ulcers, mucocutaneous disease (genital ulceration, pyoderma, etc), and vascular and neurologic manifestations (including uveitis; Figure 1).^116^ The pathogenesis of Behçet syndrome is incompletely understood, and dysregulation in both innate and adaptive immune systems has been reported. More recently, monogenic forms of mimics of Behçet syndrome have been reported and improved our understanding of more common forms of the disease.^117^ Several immune pathways have been linked with Behçet syndrome, several overlapping with SLE.

### The NF‐κB pathway

The transcriptional regulatory factor NF‐κB acts downstream of proinflammatory cytokines and other danger/stress signals. Notably, pronounced NF‐κB, tumor necrosis factor, and IL‐1 signaling have been observed in patients with “classic” and monogenic Behçet syndrome.^118^ Behçet syndrome has been linked to rare genetic variants in the *TNFAIP3* gene in 2016 with the discovery and first clinical descriptions of A20 haploinsufficiency (HA20).^119^, ^120^ *TNFAIP3*, encoding for the protein A20, is involved in regulating inflammation and immune responses by deubiquitinating regulators of NF‐κB and preventing its overactivation. HA20 is a rare genetic disorder characterized by early‐onset autoinflammatory symptoms, including recurrent fevers, oral and genital ulcers, and gastrointestinal inflammation.^120^, ^121^ Patients with HA20 most commonly exhibit a Behçet‐like clinical phenotype with symptoms such as skin lesions, arthritis, and uveitis. The condition is inherited in an autosomal‐dominant manner and typically begins in childhood in a context of dominant familial history. Colchicine has shown variable therapeutic efficacy.^121^ Single‐nucleotide polymorphisms in the *TNFAIP3* gene have been associated with various autoimmune and inflammatory diseases, highlighting the importance of this pathway in disease pathogenesis. Beyond Behçet syndrome, *TNFAIP3* variants have also been implicated in SLE, rheumatoid arthritis, Crohn disease, and other autoimmune conditions.^122^, ^123^ In a recent report from the UK cohort with juvenile SLE cohort, an additional allele in *RNASEH2A* may have shifted the phenotype of a patient with a *TNFAIP3* variant toward an SLE phenotype.^38^

Additional (ultra‐)rare variants in genes associated with the NF‐κB pathway have been identified in patients experiencing autoinflammatory episodes, including recurrent fever, oral and genital ulcers, and small‐vessel vasculitis, at least partially mimicking Behçet syndrome. Splicing and truncating mutations in *RELA*, encoding for the p65 NF‐κB subunit, have been described in patients with autosomal‐dominant mucocutaneous ulcerations. Notably, patients with *RELA* mutations fail to respond to the usual Behçet treatments and exhibit a highly inflammatory phenotype.^124^, ^125^, ^126^ Febrile attacks and small‐vessel vasculitis characteristic of Behçet syndrome may be caused by loss‐of‐function mutations in *NFKB1*, resulting in NF‐κB1 deficiency. Gain‐of‐function heterozygous variants in *RIPK1* have been described; patients display lymphadenopathy, oral lesions such as aphthosis or ulcers, abdominal pain, and hepatosplenomegaly.^127^ These three inborn errors of immunity represent <100 patients in the literature but significantly contributed to the knowledge of biologic pathways associated with Behçet syndrome.

The inhibitor of NF‐κB kinase subunit beta (*IKBKG*) gene encodes for NF‐κB essential modulator (NEMO), a key protein involved in the activation of NF‐κB.^128^ The heterozygous *IKBKG* p.Asp406Val variant has been reported in the context of incontinentia pigmenti, a rare X‐linked dominant genodermatosis in female patients. The p.Asp406Val mutation is located in the NEMO zinc finger domain, which plays a crucial role in the phosphorylation of NEMO, binding with ubiquitin, and full NF‐κB activation. Recently, *IKBKG* p.Asp406Val has also been reported in the context of Behçet syndrome, further underscoring the importance of the NF‐κB pathway in Behçet syndrome.^128^ Thus, further characterization of NF‐κB pathway components, their activation, and regulation in Behçet syndrome and related conditions may link clinical features to altered NF‐κB activation patterns and aid in the development of novel therapies (Figure 2).

### Monogenic diseases with mucocutaneous ulcerations and Behçet‐like phenotypes

The transcriptional regulator E74‐like ETS transcription factor (ELF) 4 is involved in key functions, including antiviral immune responses, immune system differentiation and regulation, inflammatory responses, and circadian rhythm. Recently, ELF4 has been linked with mucosal integrity, and deficiency of ELF4 has been identified in male patients with monogenic X‐linked diseases resembling inflammatory bowel disease and/or Behçet syndrome with oral and gastrointestinal ulcers, fever, dermatitis, arthritis, and elevated systemic markers of inflammation.^129^, ^130^ The tryptophan and aspartic acid repeat–containing protein 1, encoded by the *WDR1* gene, has been suggested to induce the disassembly of actin filaments^131^ and may therefore be involved in cytokinesis and cell motility, endocytosis, exocytosis, and mechanical cell stability.^132^ A homozygous missense variant in *WDR1* causing loss of function has been associated with a Behçet‐like clinical phenotype.^133^ Two affected Pakistani girls born to consanguineous parents displayed increased IL‐18 release, suggesting pathologic inflammasome activation and features of severe autoinflammation, including early‐onset fevers and recurrent oral and perianal ulcerations. Consequently, periodic fever, immunodeficiency, and thrombocytopenia was proposed for this condition.

Trisomy 8 is a rare chromosomal disorder. Full trisomy 8 associates with significant anomalies and commonly results in miscarriage during the first trimester. Trisomy 8 mosaicism (Warkany syndrome) is usually less severe, with a range of physical abnormalities and developmental delay.^134^, ^135^ Both forms or trisomy 8 can be associated with Behçet‐like disease phenotypes and myelodysplasia,^136^, ^137^ which is also referred to as trisomy 8–associated autoinflammatory disease. In the absence of a defined molecular therapeutic target, hematopoietic stem cell transplantation has been tried (when indicated for refractory myelodysplasia) and was successful in relation to Behçet‐like symptoms (Figure 2).^138^

## Discussion

The study of rare and ultra‐rare genetic variants has significantly advanced our understanding of common autoimmune/inflammatory diseases as well as normal immune function. Elucidation of genetic and molecular mechanisms underlying rare inflammatory conditions has already resulted in target‐directed and highly effective therapies, ultimately improving patient outcomes (Figure 2).^45^ Here, we illustrate the complex genetic architecture of monogenic defects that can drive distinct clinical phenotypes such as SLE or Behçet syndrome. The underlying pathways involve innate or adaptative immune responses with some genetic defects, namely like HA20 and *RELA* mutations, promoting both.^121^, ^122^, ^124^ Although a recent publication suggested additional alleles contribute to differential phenotype expression in patients with *TNFAIP3* variants (and other genetically defined diseases), the exact molecular underpinnings of incomplete genotype to phenotype correlation remains unclear.^38^ Notably, environmental impact and associated epigenetic patterns have also recently been suggested as contributors to phenotypic variability in defined genetic diseases.^139^ In patients with AGS and the *RNASEH2B* p.A177T genotype, DNA methylation of IFN‐associated genes varied between patients with “mild” versus “severe” neuropsychiatric involvement, and DNA methylation scores allowed the prediction of neurologic outcomes.^139^ This suggests that studies in (ultra‐)rare genetic diseases may be a tool to gain a better understanding of host to environment interactions and their contribution to chromatin structure of autoimmune and inflammatory disease. Understanding the interplay among genetic variation, chromatin composition, and clinical phenotypes will result in the development of tools for molecular risk assessment and patient stratification toward individualized treatment and care.

Targeted treatments have been used in several systemic autoimmune/inflammatory diseases, including (but not limited to) IL‐1 blocking strategies in cryopyrin‐associated periodic syndromes and otherwise treatment‐refractory FMF.^140^, ^141^ It becomes increasingly clear that, among patients with SLE, those with biallelic mutations in C1q should not be treated the same way as those with biallelic mutations in *TREX1*, even if they exhibit similar clinical phenotypes. Taken together, personalized precision medicine concepts that have first been applied in cancer treatment become increasingly available for patients with autoimmune/inflammatory diseases. However, phenotypical assessment based on clinical features alone cannot safely provide information on the molecular phenotype and treatment targets (as suggested by aforementioned HA20 being associated with SLE and Behçet syndrome).^121^ Therefore, genetic screening will likely become routine in the diagnosis and care of SLE and Behçet syndrome in the coming years, especially when the disease presents early, displays atypical features, and/or occurs in multiple family members.

Through the ongoing identification and characterization of “new” inborn errors of immunity, the fields of clinical immunology and rheumatology become increasingly complex, which warrants interdisciplinary collaboration involving rheumatologists, immunologists, infectious disease specialists, and more. Beyond the two disease entities discussed here, numerous other early‐onset inflammatory diseases have been associated to monogenic defects. Among the most characterized are sarcoidosis inflammatory bowel diseases, vasculitis, and systemic sclerosis (Table 1). Interestingly, Still disease and juvenile dermatomyositis (JDM) have not been commonly associated to single‐gene mutations, except in patients with extremely rare *LACC1* deficiencies or JDM mimics with chronic atypical neutrophilic dermatosis with lipodystrophy and elevated temperature (CANDLE) genotypes.^161^, ^162^, ^163^ This suggests that inflammatory myosis or juvenile idiopathic arthritis may be less driven by genetic factors when compared to other rheumatic inflammatory diseases.

**Table 1:** Main monogenic causes of systemic inflammatory diseases*

Disease/gene	Protein	Inheritance	Clinical features	Reference
**IBDs**				
*NOD2*	NOD2	AD	Blau syndrome, camptodactyly, uveitis, arthritis	142
*CYBB*	Gp91phox	X linked	Infections, aphtes	143
*IL10*, *IL10RA*, *IL10RB*	IL‐10, IL‐10RA, IL‐10RB	AR	Folliculitis, recurrent respiratory disease	144, 145
*NFAT5*	NFAT5	AD	Recurrent infections	146
*TGFB1*	TGFB1	AR	Recurrent viral infection, microcephaly, encephalopathy	147
*RIPK1*	RIPK1	AR	Recurrent infections, polyarthritis	148
*ELF4*	ELF4	X linked	Behçet disease, fever	129, 149
*XIAP*	XIAP	X linked	Hepatosplenomegaly	150
**Sarcoidosis/systemic granulomatosis**				
*RAG1/2*, *PRKDC*	RAG1, RAG2, DNA‐PK	AR/AD	Opportunistic infections, T^−^B^−^NK^+^SCID	151, 152
Other *SCID* genes	Other T cell–related genes	AR	T^−^B^−^NK^−^SCID	151, 152
*CTLA4/LRBA*	CTLA4/LRBA	AD/AR	Autoimmune cytopenia, lymphadenopathy, hypergammaglobulinemia, IBD	153, 154
*NOD2*	NOD2	AD	Blau syndrome, camptodactyly, uveitis, arthritis	142, 155
**Polyarteritis nodosa**				
*ADA2*	ADA2	AR	Early‐onset recurrent ischemic stroke, fever, aplastic anemia	156, 157
**Systemic sclerosis**				
*STAT4*	STAT4	AD	Pansclerotic morphea, cytopenia, hypogammaglobulinemia	158
*ATAD3A*	ATAD3A	AR/AD	Developmental delay, hypotonia	159
**ANCA‐associated vasculitis**				
*STING1*, *COPA*, *DNASE1L3*	STING,COPA, DNASE1L3	AD, AD, AR	Lung hemorrhage, interstitial lung disease, kidney disease ± lupus features	66, 67, 160

* AD, autosomal dominant; ADA, adenosine deaminase; ANCA, antineutrophil cytoplasmic antibody; AR, autosomal recessive; ATAD, ATPase family AAA domain; COPA, coatomer subunit alpha; ELF, E74‐like ETS transcription factor; IBD, inflammatory bowel disease; IL, interleukin; LRBA, lipopolysaccharide‐responsive beige‐like anchor protein; NK, natural killer; NOD, nucleotide‐binding oligomerization domain; PK, protein kinase; RAG, recombination‐activating gene; RIPK, receptor‐interacting serine/threonine PK; SCID, severe combined immunodeficiency; STING, stimulator of interferon genes; T^−^B^−^NK^+^SCID: SCID with T and B cell deficiency and relatively normal NK cells; T^−^B^−^NK^−^SCID, SCID with T, B, and NK cell deficiency; TGF, transforming growth factor; XIAP, X‐linked inhibitor of apoptosis protein.

A new area of interest in recent years is somatic mutations.^164^ Somatic mutations in STING‐associated vasculopathy with onset in infancy has been reported in siblings with early‐onset polyarthritis, chilblain lupus, and interstitial pneumopathy.^165^ Additionally, X‐linked VEXAS (V: vacuoles; E: E1 ubiquitin conjugating enzyme encoded UBA1; X: X chromosome; A: autoinflammatory; S: somatic mutations) syndrome represents one of the most frequent autoinflammatory syndromes that typically presents during the second half of life, contributing to the understanding of rare diseases such as relapsing polychondritis.^166^ Considering the tissue‐specific expression of genes and the underrecognized field of somatic mutations, it is anticipated that many other rare monogenic variants will be identified in the field of autoinflammatory diseases.

## Conclusions

The insights gained from studying rare large‐effect‐size variants enhanced our understanding of immune function in health and disease. It explained the pathogenesis of many rare inflammatory diseases and provided the foundation for personalized medicine, in which treatments are tailored to the genetic profile of individual patients. Molecular genetic studies in patients with inflammatory diseases alongside collaborative structured collection and monitoring of patients will identify and characterize (new and already known) genetically determined diseases and further improve our understanding of rare and common immune‐mediated diseases.

## AUTHOR CONTRIBUTIONS

All authors contributed to at least one of the following manuscript preparation roles: conceptualization AND/OR methodology, software, investigation, formal analysis, data curation, visualization, and validation AND drafting or reviewing/editing the final draft. As corresponding author, Drs Belot and Hedrich confirm that all authors have provided the final approval of the version to be published and take responsibility for the affirmations regarding article submission (eg, not under consideration by another journal), the integrity of the data presented, and the statements regarding compliance with institutional review board/Declaration of Helsinki requirements.

## Supporting information


**Disclosure Form**:
